# Hand, Foot and Mouth Disease: Changing Indian Scenario

**DOI:** 10.5005/jp-journals-10005-1171

**Published:** 2012-12-05

**Authors:** Prasanna Kumar Rao, KM Veena, H Jagadishchandra, Sham S Bhat, Shishir Ram Shetty

**Affiliations:** Reader, Department of Oral Medicine and Radiology, Yenepoya Dental College, Yenepoya University, Mangalore, Karnataka, India e-mail: drjpkrao@gmail.com; Professor, Department of Oral Medicine and Radiology, Yenepoya Dental College, Yenepoya University, Mangalore, Karnataka, India; Professor, Department of Oral and Maxillofacial Surgery, Yenepoya Dental College, Yenepoya University, Mangalore, Karnataka, India; Vice Principal, Department of Pedodontics and Preventive Dentistry Yenepoya Dental College, Yenepoya University, Mangalore Karnataka, India; Assistant Professor, Department of Oral Medicine and Radiology AB Shetty Memorial Institute of Dental Sciences, Mangalore Karnataka, India

**Keywords:** Hand, foot and mouth disease, Coxsackievirus infection, Enterovirus, Vesicular stomatitis with exanthema

## Abstract

Hand, foot and mouth disease usually affect infants and children. Although seen worldwide, it is not common in India. It is moderately contagious and is spread through direct contact with the mucus, saliva, or feces of an infected person. It typically occurs in small epidemics, usually during the summer and autumn months. The incidence of hand, foot and mouth disease has recently been on the rise in India due to the probable mass immunization programs. This report describes a case of hand foot and mouth disease from Mangalore, South India.

**How to cite this article:** Rao PK, Veena KM, Jagadishchandra H, Bhat SS, Shetty SR. Hand, Foot and Mouth Disease: Changing Indian Scenario. Int J Clin Pediatr Dent 2012;5(3):220-222.

## INTRODUCTION

The most common strains causing hand, foot and mouth disease (HFMD) are Coxsackie A16, a type of enterovirus, which mostly affects children below 10 years of age.^1^ The usual incubation period is 3 to 7 days. Early symptoms are likely to be fever often followed by a sore throat. Loss of appetite and general malaise may also occur. Between 1 and 2 days after the onset of fever, painful sores (lesions) may appear in the mouth or throat. A rash may become evident on the hands, feet, mouth, tongue, inside of the cheeks and also the buttocks, knees and elbow. Oral lesions appear as vesicles, which rapidly ulcerate producing multiple small superficial ulcers with erythematous halos. The ulcers are usually seen on the tongue, palate, buccal mucosa, gums and lips. Oral ulcers cause discomfort, making oral feeding difficult.^2^

## CASE REPORT

**Figs 1A to E:**
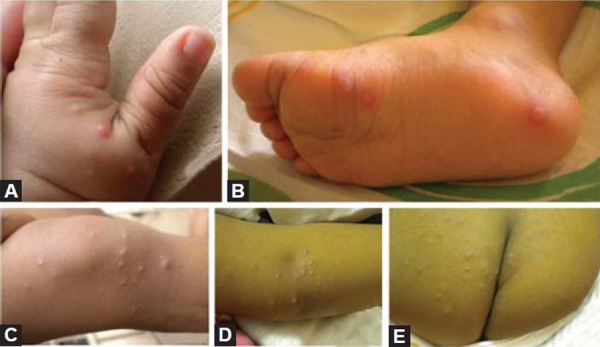
Multiple eruptions over the hand, feet, knee, elbow and buttocks

**Fig. 2:**
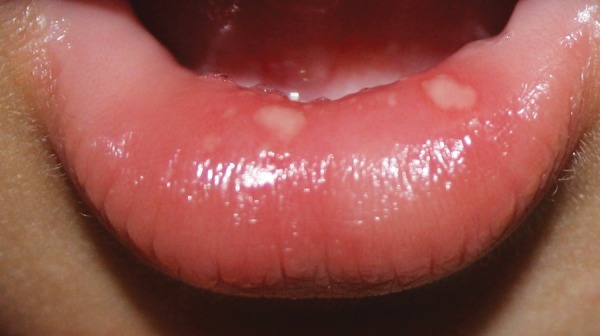
Oral ulcers on the labial mucosa of lower lip

**Figs 3A to E:**
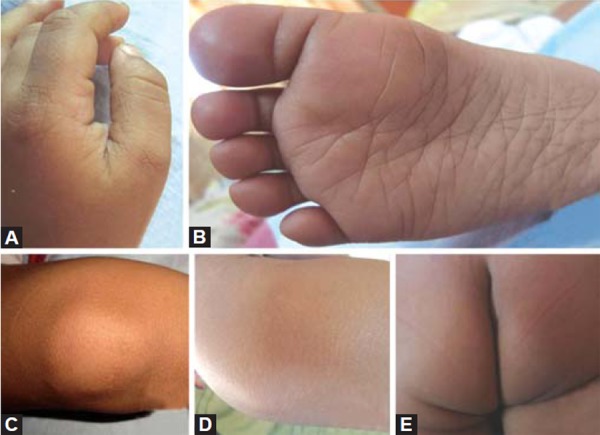
Complete healing of the lesions over the hand, feet, knee, elbow and buttocks

**Fig. 4:**
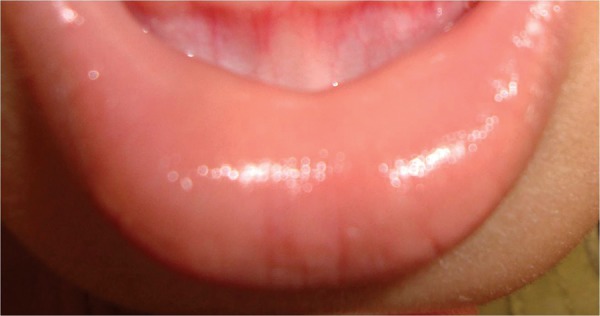
Healing of oral ulcers without any scaring

A 1-year-old male child was referred from department of pediatrics for opinion regarding oral ulcerations. The child was brought to the department of pediatrics by his parents with complaints of fever and skin rashes for a period of 3 days. On general examination there were multiple eruptions over the hand, feet, knee, elbow and buttocks (Figs 1A to E). The skin eruptions were around 2 mm in diameter and filled with clear fluid. The oral ulcers were distributed mainly on the labial mucosa of lower lip (Fig. 2). The ulcers were around 2 mm in diameter, irregular in shape, covered with a reddish halo and had yellowish base. The lower lip was edematous. Based on the clinical features and pediatric consultation a provisional diagnosis of HFMD was made. Since the disease was self-healing no specific treatment for oral ulcers was given. However, the pediatricians prescribed ointment aloe vera 10% lotion to be applied three times over the eruptions and syrup mefenamic acid 100 mg syrup one TSP three times daily for 5 days. The patient was revived after 20 days. Complete healing of the lesions was noted in all previously reported sites (Figs 3A to E). On examination of the oral mucosa complete healing was noticed without any scaring (Fig. 4).

## DISCUSSION

HFMD also known as vesicular stomatitis with exanthema in literature.^3^ Coxsackievirus infection is highly contagious. During epidemics, the virus is spread by horizontal transmission. Initial viral implantation in the buccal and ileal mucosa is followed by spread to lymph nodes within 24 hours. Oral lesions begin as erythematous macules that evolve into 2 to 3 mm vesicles on an erythematous base. The vesicles may involve the palate, buccal mucosa, gingiva, lip and tongue. The vesicles are rarely observed because they rapidly become ulcerated. They are painful and may interfere with mastication and feeding. In 44% of the cases, tongue involvement is reported.^4^ Viremia rapidly ensues, with spread to the oral mucosa and skin. After a week, neutralizing antibody levels increase and the virus is eliminated.^5^

There is no normal enteric virus flora. Usually only one type of enterovirus multiplies within the intestine of an individual at any given time. Polio vaccination has eliminated polio viruses from the gut, thereby increasing the chances of coxsackie viral and echoviral infections. It is possible that the emergence of HFMD in India may be related to the mass polio vaccination. However, a firm conclusion can be made in this regard only after studying a large number of cases of HFMD over a period of time.^6^ It is uncommon in adults, but those with immune deficiencies are very susceptible. HFMD is not to be confused with foot- and-mouth disease (also called hoof-and-mouth disease), which is a disease affecting sheep, cattle and swine, and which is unrelated to HFMD.

The first major outbreak of HFMD occurred in Sarawak, Malaysia in 1997 in the Asia Pacific region.^7^ The largest outbreak of HFMD occurred in eastern part of India in 2007, where about 38 cases of HFMD in and around Kolkata was reported.^8^

Complications like dehydration, meningoencephalitis, myocarditis, pulmonary edema, and death occasionally occurs in children with HFMD.^9^ Viral meningitis causes fever, headache, stiff neck or back pain. Some patients may need to be hospitalized for a short time. Complications from the virus infections that cause HFMD are not common, but if they do occur, medical care should be given.

Patients may first consult a dermatologist due to prominent skin manifestations of HFMD. Oral lesions of HFMD can be easily misdiagnosed as aphthous ulcers, varicella or herpangina. However, varicella rarely presents with oral lesions and the skin lesions are more concentrated on the trunk, rarely affecting the palms and soles. Herpangina is a viral infection of children caused by a type A coxsackie virus which presents with similar types of oral ulcers but are more extensive involving the tonsils, pharyngeal mucosa, soft palate and the posterior part of buccal mucosa.^2^

Treatment includes the topical application of anesthetics and viscous lidocaine or diphenhydramine for painful oral ulcers. Antipyretics may be used to manage fever, and analgesics may be used to treat arthralgias. Low-level laser therapy has also shortened the duration of painful oral ulcers.^10^

## CONCLUSION

Increased awareness about vaccination in a developing nation like India and vaccinization program at the grass root levels have eradicated certain lethal diseases. At the same time viral disorders previously unreported in Indian population are now being diagnosed more often. Thus, pediatric dentist or a general dental practitioner needs to be aware of such disease for timely diagnosis and prompt treatment.
